# Minimally invasive non-surgical vs. surgical approach for periodontal intrabony defects: a randomised controlled trial

**DOI:** 10.1186/s13063-019-3544-8

**Published:** 2019-07-27

**Authors:** L. Nibali, V. Koidou, S. Salomone, T. Hamborg, R. Allaker, R. Ezra, L. Zou, G. Tsakos, N. Gkranias, N. Donos

**Affiliations:** 1 Centre for Oral Immunobiology and Regenerative Medicine, Centre for Oral Clinical Research, Institute of Dentistry, Queen Mary University of London (QMUL), London, UK; 2 Pragmatic Clinical Trials Unit, Centre for Primary Care and Public Health, Queen Mary University of London (QMUL), London, UK; 3 Department of Epidemiology and Public Health, University College London (UCL), London, UK; 4 Periodontology Unit, Centre for Host Microbiome Interactions, Faculty of Dentistry, Oral & Craniofacial Sciences, King’s College London, London, UK

**Keywords:** Periodontitis, Intrabony defect, Minimally invasive, Quality of life, Bone

## Abstract

**Background:**

Periodontal intrabony defects are usually treated surgically with the aim of increasing attachment and bone levels and reducing risk of progression. However, recent studies have suggested that a minimally invasive non-surgical therapy (MINST) leads to considerable clinical and radiographic defect depth reductions in intrabony defects. The aim of this study is to compare the efficacy of a modified MINST approach with a surgical approach (modified minimally invasive surgical therapy, M-MIST) for the treatment of intrabony defects.

**Methods:**

This is a parallel-group, single-centre, examiner-blind non-inferiority randomised controlled trial with a sample size of 66 patients. Inclusion criteria are age 25–70, diagnosis of periodontitis stage III or IV (grades A to C), presence of ≥ 1 ‘intrabony defect’ with probing pocket depth (PPD) > 5 mm and intrabony defect depth ≥ 3 mm. Smokers and patients who received previous periodontal treatment to the study site within the last 12 months will be excluded. Patients will be randomly assigned to either the modified MINST or the M-MIST protocol and will be assessed up to 15 months following initial therapy. The primary outcome of the study is radiographic intrabony defect depth change at 15 months follow-up. Secondary outcomes are PPD and clinical attachment level change, inflammatory markers and growth factors in gingival crevicular fluid, bacterial detection, gingival inflammation and healing (as measured by geometric thermal camera imaging in a subset of 10 test and 10 control patients) and patient-reported outcomes.

**Discussion:**

This study will produce evidence about the clinical efficacy and potential applicability of a modified MINST protocol for the treatment of periodontal intrabony defects, as a less invasive alternative to the use of surgical procedures.

**Trial registration:**

ClinicalTrials.gov, NCT03797807. Registered on 9 January 2019.

## Background

Periodontal diseases are inflammatory conditions that affect the supporting apparatus of the teeth. Periodontitis and its non-destructive partner condition, gingivitis, are collectively one of the most prevalent inflammatory conditions of humanity [1]. In 2010, severe periodontitis was estimated to be the sixth most prevalent disease in the world, with a prevalence of 11.2%, gradually increasing with age [1]. In periodontitis, the presence of subgingival plaque biofilms in susceptible individuals determines an inflammatory reaction, leading to loss of the supporting connective tissue and alveolar bone. Periodontitis is now classified into different stages (I to IV), based on disease severity, and into grades (A to C), based on risk of progression [2]. Periodontal osseous destruction can result in horizontal or vertical bony defects, depending on the direction and extent of the apical propagation of the plaque-induced lesion [3]. The treatment of periodontitis involves a non-specific reduction of the bacterial load below the gingival margin [4], which can be achieved by oral hygiene instructions and non-surgical periodontal therapy (NSPT). More advanced cases need surgical treatment or extractions. The overall objective of the treatment is the elimination of periodontal inflammation through disruption of the subgingival biofilm, with reduction of gingival probing pocket depth (PPD) and clinical attachment loss (CAL), resulting in reduced risk of disease progression [5–7].

Periodontal vertical bony defects (intrabony defects) have been associated with a higher risk of progression and eventually tooth loss [8]. Therefore, they are considered sites requiring therapy, often beyond NSPT. The treatment of intrabony defects has gradually evolved from radical surgical elimination of the defect by removal of some of the adjacent healthy supportive or non-supportive bone [9] to more conservative surgical approaches [10] and then to regenerative surgical procedures resulting in regeneration of periodontal attachment measurable clinically, radiographically and histologically [11]. However, even these less invasive surgeries are associated with potential morbidity and high costs due to the use of bone graft and barrier materials, and they are not always predictable [12]. More recently, minimally invasive surgical therapy (MIST), modified minimally invasive surgical therapy (M-MIST) and single-flap approach [13] techniques were introduced, adapting regenerative procedures to the principles of minimally invasive surgery. Results of studies using these techniques suggest that the use of biomaterials may not be so crucial for obtaining periodontal regeneration [14]. A recent consensus report of the American Academy of Periodontology considers surgical intervention still the treatment of choice for intrabony defects [15].

Pushing the boundaries of minimal invasiveness, a minimally invasive non-surgical therapy (MINST) protocol has recently been proposed for the treatment of intrabony defects [16]. A recent retrospective analysis has revealed a reduction in bony defect of approximately 3 mm for cases treated with MINST in non-smokers [17]. This improvement seems to be stable at least up to 5 years after treatment, despite no surgical intervention [18]. The effect of MINST may be mediated by improved blood flow and stable blood clot in the intrabony defect. However, no study to our knowledge has tested the efficacy of MINST compared with traditional NSPT followed by surgical intervention (M-MIST) in the osseous healing of intrabony defects.

The primary objective of this study is to compare the efficacy of a further refinement of the MINST approach (modified MINST) with a surgical approach (M-MIST) for the treatment of intrabony defects. Secondary objectives are to (1) compare radiographic intrabony angle change from before to after treatment and between groups; (2) investigate the association between local factors (gingival blood flow, gingival crevicular fluid [GCF] concentration of inflammatory markers, lipids and growth factors, bacterial presence) and intrabony defect depth healing; and (3) investigate the association between patient-based demographic and health-related factors (age, gender, medical history, body mass index [BMI]) and intrabony defect depth healing.

## Methods/design

This is a parallel-group, single-centre, examiner-blind, non-inferiority randomised controlled trial (RCT) to compare the effect of the following interventions in the healing of periodontal intrabony defects in 66 patients with chronic periodontitis (CP):

1. A novel non-surgical treatment protocol (modified MINST)
2. A surgical protocol (M-MIST).

### Inclusion criteria

The following criteria will be required for inclusion in the study:

1. Age 25–70
2. Diagnosis of ‘periodontitis’ stage III or IV (grades A to C)
3. Presence of ≥ 1 ‘intrabony defect’ (PPD, > 5 mm with intrabony defect depth ≥ 3 mm at screening radiograph)
4. Signed informed consent.

### Exclusion criteria

The exclusion criteria are as follows:

1. Smoking (current or in past 5 years)
2. Medical history including diabetes or hepatic or renal disease or other serious medical conditions or transmittable diseases
3. History of conditions requiring prophylactic antibiotic coverage prior to invasive dental procedures
4. Anti-inflammatory or anticoagulant therapy during the month preceding the baseline exam
5. Systemic antibiotic therapy during the 3 months preceding the baseline exam
6. History of alcohol or drug abuse
7. Self-reported pregnancy or lactation
8. Other severe acute or chronic medical or psychiatric condition or laboratory abnormality that according to the investigator may increase the risk associated with trial participation
9. Periodontal treatment to the study site within the last 12 months.

### Study design/plan

All patients will be recruited from the Restorative and Periodontal new patient clinics at Barts and The London Dental Hospital, where potentially suitable new periodontitis patients will be informed about the study. Then a member of the research team will approach potentially interested patients, will provide more information about the study procedures together with the benefits and risks of participation and will give suitable patients the patient information sheet, which they will be advised to read carefully. Potential participants will be informed that they will be allowed to withdraw their participation at any stage of the study. The patients will then be contacted within 1 week to enquire about their willingness to take part in the study and to give them the opportunity to ask any questions about it. Upon agreeing to partake, they will be offered a baseline appointment. Written informed consent (including clinical procedures and collection of study samples) will be obtained before enrolment. Informed consent will follow the Barts and The London Dental Hospital and Queen Mary University of London (QMUL) standard operating procedures and will be conducted by staff trained in taking consent.

### Study visits

**Fig. 1:**
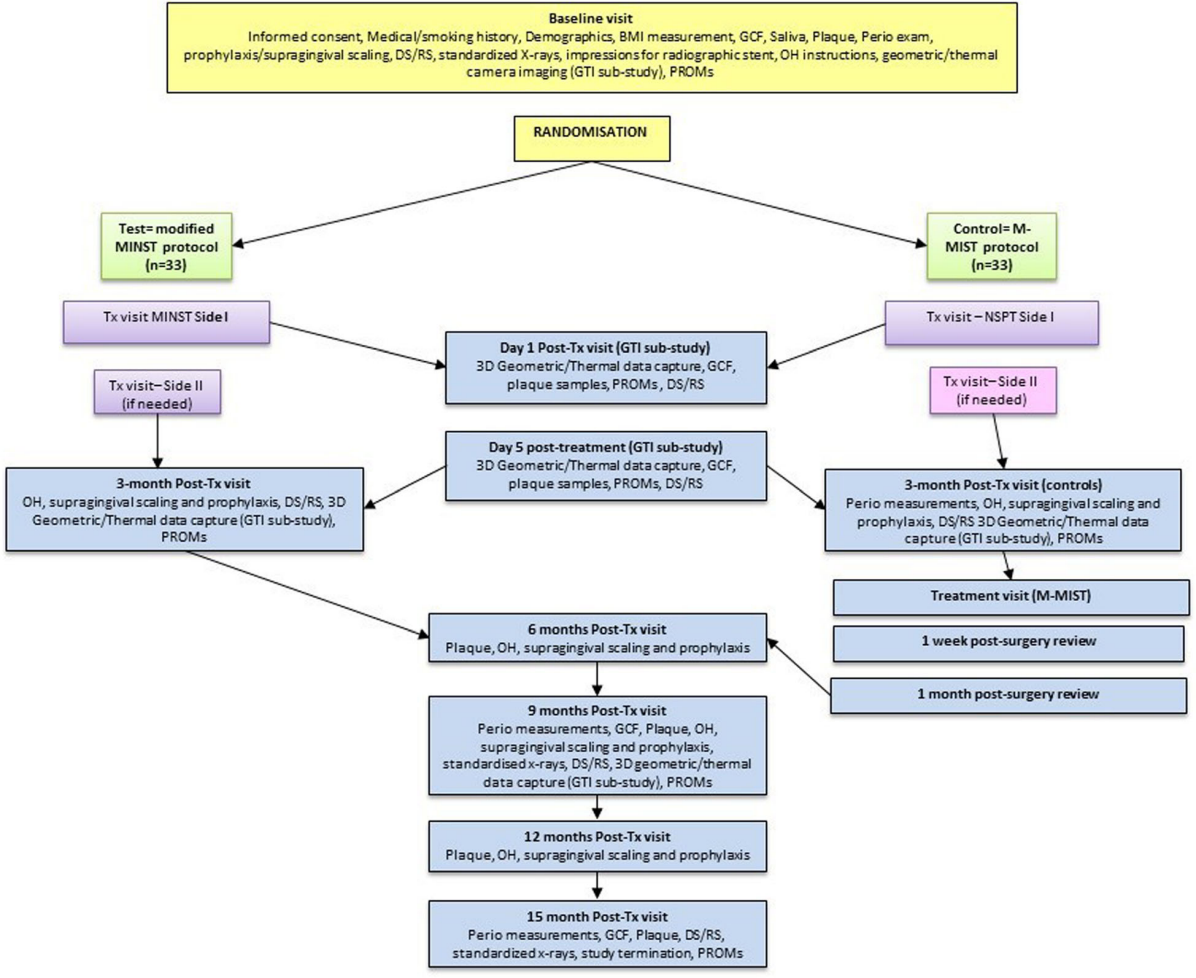
Flowchart of study plan and visits

**Fig. 2:**
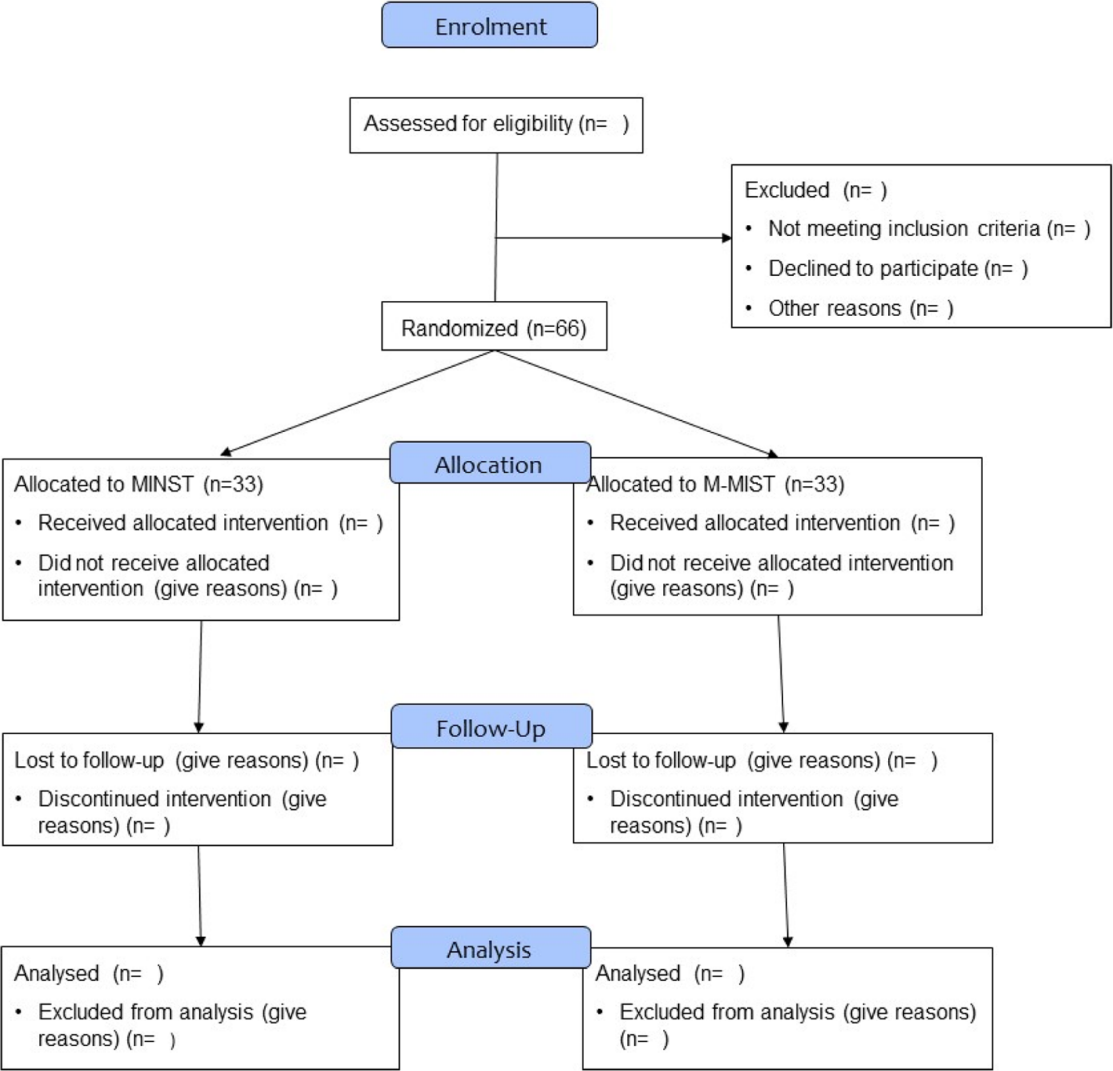
CONSORT flowchart

Each subject will attend between 8 and 13 study visits over a period of approximately 16 months (see Table 1 for the study visit schedule and procedures). All the visits will take place in the clinics of Barts and The London Dental Hospital. The schedule of assessments is also presented in Fig. 1 (flowchart of study plan and visits) and in Fig. 2 in the form of a Consolidated Standards of Reporting Trials (CONSORT) diagram.

**Table 1:** Schedule of study visits and procedures

Protocol procedures	Visit 1	Visit 2	Visit 2a	Visit 2b	Visit 3	Visit 4	Visit 4a	Visit 4b	Visit 4c	Visit 5	Visit 6	Visit 7	Visit 8
	Baseline	Side I treatment	1day post-treatment	5 days post-treatment	Side II treatment	3 months post- treatment	Surgical treatment	1 week suture removal	1 month review	6 months post- treatment	9 months post- treatment	12 months post- treatment	15 months post- treatment
	Week 0		GTI subgroup only	GTI subgroup only			Only subjects in MIST group	Only subjects in MIST group	Only subjects in MIST group				
Verification inclusion/exclusion, informed consent	x												
Medical/dental history & updates Demographics	x	x	x	x	x	x	x	x	x	x	x	x	x
Height, weight, waist circumference	x												
3D facial imaging	x		x	x		x		x	x		x		
Periodontal measurements (PPD, gingival recession, bleeding on probing, mobility and furcation involvement)	x					x				x	x		x
Plaque assessment	x					x				x	x	x	x
PROMs	x		x	x		x		x			x		x
Early healing index	x							x					
Standardised X-ray		x									x		x
GCF collection	x		x	x		x					x		x
Subgingival plaque collection/saliva	x		x	x		x					x		x
Debridement	x					x				x		x	
Impression for radiographic stent	x												
Dentine/root sensitivity measurements	x		x	x		x					x		x

### Study procedures

#### Medical history

A complete medical history will be obtained at the screening visit, including demographic background information and dental status information. This will be reviewed and updated throughout the study. All concomitant medications, procedures and adjunctive product use will be monitored and recorded throughout the study. Tobacco use history will be recorded at baseline based on self-report. BMI will be calculated after measuring the participant’s height and weight.

#### Clinical periodontal assessment

The following periodontal measurements will be taken by the calibrated examiner (author VK) at six sites/tooth using a manual University of North Carolina (UNC-15) periodontal probe (Hu-Friedy, Chicago, IL, USA): dichotomous full-mouth plaque score (FMPS) [19], full-mouth PPD, recession of the gingival margin from the cemento-enamel junction (CEJ), dichotomous 6-point full-mouth bleeding score (FMBS) on probing [19], tooth mobility [20] and furcation involvement with a Nabers probe (Hu-Friedy) [21]. Clinical attachment level (CAL) will be calculated as PPD+ recession. The early healing index [22] will be measured for controls 1 week after surgery. Dentine/root sensitivity (DS/RS) will be assessed on the tested intrabony tooth and on the two neighboring teeth, following isolation using cotton wool rolls, by using an evaporative/thermal stimulus from a 1-s blast of air from a dental unit syringe at 40–65 psi (19–23 °C) directed perpendicular to the tooth surface at a distance of 0.5–1 cm to the exposed root surface (evaporative examination) [23]. Following the air blast, the subject will be given a visual analogue scale (VAS) form to complete each assessment. The VAS is scored from 0 = no pain to 10 = extreme pain, and the subject can indicate the degree of discomfort by indicating either a numerical value from 0 to 10 or by marking vertically across the VAS.

#### Repeatability

Following initial training, for a repeatability exercise the examiner and back-up examiner will perform repeated examinations on 10 subjects for PPD and recession with at least 15 min separation. Upon completion of all measurements, intra-examiner repeatability for PPD measurements will be assessed. In order to test inter-examiner repeatability, at least 10 subjects will be probed twice (once by each examiner). The reproducibility of the examiner will be tested using the Bland–Altman approach [24] and by calculating Lin’s concordance correlation coefficient [25]. A coefficient of repeatability less than ±2 mm in 95% of the cases is considered acceptable. If this target is not achieved, further examiner assessment and training will be carried out before a new reproducibility exercise is performed.

#### Clinical photographs

Clinical photographs and videos of completed procedures will be taken in some of the study visits for better documentation of cases and will be anonymised and stored in the study database.

#### Radiographic analyses

Standardised radiographs of selected study sites will be taken at baseline and at the 9 and 15 months follow-up visits using the parallel technique with a customised holder and an occlusal platform, which will allow a cold-cure acrylic resin occlusal registration to be made (bite index), facilitating relocation of the holder and preserving the projection geometry in subsequent radiographs. Aluminium step wedges will be used as a densitometric reference in an attempt to minimise errors due to variation in exposure time and/or film processing which may result in false positive analysis [26]. The linear radiographic measurement analysis will be carried out by a single calibrated examiner using semi-automated radiographic software with specific landmarks, as described by Nibali et al. [27]. In brief, horizontal and vertical bone loss of the intrabony defects will be measured after identifying the following landmarks in periapical radiographs: CEJ on the tooth with the intrabony defect, most coronal part of the alveolar bone and most apical part of the alveolar bone crest, where the periodontal ligament space was judged to retain its normal width. The radiographs will be analysed by the same trained and calibrated examiner in a masked, random order with the use of a computer software measurement tool (Emago®, Oral Diagnostic Systems, Amsterdam, the Netherlands).

#### Test procedure (MINST)

Based on our previous study [17], a modified MINST protocol for this study consists of the following:

- Local anaesthesia by infiltration without adrenaline in the study site (not intrasulcular anaesthesia)
- Thorough debridement of the root surface up to the bottom of the periodontal pocket under local anaesthesia
- Attempt to minimise the trauma to the soft tissues and especially to the papilla, using a subpapillary access for debridement (trying not to touch the most coronal part of the interdental papilla)
- Use of exclusively piezo-electric devices with specific thin and delicate tips
- Deliberately avoid ‘smoothing’ the root surface or performing gingival curettage
- Use of 3–4× magnification loupes
- Attempt to stimulate the formation of a stable blood clot, by natural filling of the intrabony defect with blood following debridement (no use of any subgingival rinses).

This modified protocol differs slightly from the published protocol [17] in the use of a subpapillary access; exclusively piezo-electric devices (no curettes); the specific use of anaesthesia without adrenaline and no intrasulcular anaesthesia; and the first subgingival probing at 6 months post-treatment (rather than at 3 months). These slight refinements from the earlier studies [17] aim to further reduce tissue trauma and to stimulate a stable blood clot for optimal healing. This modified MINST protocol will be applied to the intrabony sites and all other affected sites in test patients. The same treatment protocol is currently being tested in a separate single-arm prospective multicentre study (ClinicalTrials.gov reference NCT03741374).

#### Control procedure (M-MIST)

This will be performed as described in the literature [14, 28]:

1. Scaling and root planing using conventional ultrasonic tips and curettes under local anaesthesia
2. Periodontal re-evaluation and charting 3 months later
3. In case of residual PPD > 5 mm, surgical access with the following protocol:
  - Ideally, the experimental sites will be accessed with the M-MIST technique (elevation of buccal flap only with simplified or modified papilla preservation incisions, no papilla elevation) and carefully debrided.
  - When a defect wraps around the lingual aspect of a tooth and M-MIST is not applicable, elevation of the interdental soft tissues becomes necessary and MIST becomes the preferred approach. This consists of elevation of small buccal and lingual flaps (with modified papilla preservation incision in case of interproximal space width of at least 2 mm or otherwise simplified papilla preservation [29].
  - When the position of the residual buccal/lingual bony wall(s) is very deep and difficult or impossible to reach with the above-described minimal incision of the defect-associated interdental space, the flap(s) will be further extended mesially or distally involving one extra interdental space to obtain a larger flap reflection [29].

Should the FMPS not reach the threshold below 25% before surgery, additional oral hygiene (OH) instructions will be given before proceeding with the surgical intervention. If after three additional OH sessions, the FMPS is still not below 25%, the patient will be exited from the study as not considered suitable for surgery [30]. Measurements will be taken during surgery to characterise the defect anatomy (number of walls, depth and width). No regenerative material/devices will be applied. Flaps will be sutured with modified internal mattress sutures (and single interrupted sutures if necessary).

#### Need for further treatment during and following study completion

Treatment to sites other than the selected ‘intrabony site’ throughout the study will consist of subgingival debridement (as allocated by randomisation in test and control patients) and supportive therapy as per protocol. The need for other surgical interventions will be reviewed following the 15 months follow-up. Should deterioration be detected during the study in any sites, where urgent need for surgery or antibiotics is needed, this will be carried out in additional visits during the study and will be documented in the Case Report Forms (CRFs). Following the 15 months follow-up, the possible need for further treatment will be assessed based on residual PPDs > 5 mm [4, 31].

#### Gingival crevicular fluid (GCF) sampling

An ‘intrabony site’ (IS) and a ‘comparison site’ (CS) will be chosen for sampling for each patient among buccal sites. The IS is selected according to the inclusion definitions above. In case of multiple ISs per patient, the site with deepest PPD will be chosen. The CS will be a site with PPD < 4 mm that is not bleeding on probing at the screening visit. Samples of GCF will be collected from the selected IS and CS (for both test and control subjects) at baseline, at the 3 months visit and at the 9 and 15 months follow-ups, prior to periodontal probing to avoid contamination by blood. In the 20 randomly selected subjects taking part in the ‘GTI substudy’, additional GCF sampling will be conducted at day 1 and day 5 visits following initial treatment (for both test and control subjects). Saliva will be removed from the supragingival area using a saliva ejector and cotton rolls, with care taken not to touch the gingival margin area; supragingival plaque, if present, will be removed using a curette to prevent saliva and/or plaque contamination. GCF will be collected for 30 s using methylcellulose strips carefully placed gently at the entrance of the sulcus until slight resistance is felt. GCF volume will be routinely estimated by Periotron (OraFlow Inc., Hewlett, NY, USA). GCF will be immediately extracted in acidic buffer to better preserve inflammatory mediators of periodontal disease from breakdown and/or oxidative processes, which occur to a major extent on the paper strips during storage [32]. GCF samples will then be immediately placed into small conic vials and stored at − 80 °C until time of analysis. Samples will then be processed at the Blizard Institute, Barts and the London School of Medicine and Dentistry, where immunoassay of inflammatory molecules (including, for example, levels of cytokines) and growth factors (for example, bone morphogenetic protein-2 [BMP-2]) in the GCF will be performed. The QMUL Centre for Oral Clinical Research (COCR) and the Blizard’s local standard operating procedures, working practices and risk assessments will be followed to ensure the integrity and viability of all samples to be anonymised, labelled, stored and transferred.

#### Subgingival plaque sampling

Following GCF collection, the IS and CS will have samples of subgingival plaque collected (for both test and control subjects) from the palatal/lingual aspect. Plaque samples will be collected from the selected IS and CS (for both test and control subjects) at baseline, at the 3 months visit and at the 9 and 15months follow-ups. In the 20 randomly selected subjects taking part in the GTI substudy, additional plaque sampling will be conducted at day 1 and day 5 visits following initial treatment (for both test and control subjects). Ahead of the sampling procedure, the supragingival plaque will be carefully removed and the site isolated with cotton rolls and gently dried. A sterile curette will then be inserted to the bottom of the pocket and removed after a single stroke, and the content will be placed in a test tube containing reduced transport fluid until time of analysis. Plaque samples will be analysed using next generation marker DNA sequencing to characterise the subgingival microbiota in order to identify and determine the levels of key periodontal bacterial pathogens and microbial community-wide changes in sites treated with both test and control protocols.

#### Geometric thermal imaging (GTI) substudy

A random sample of 20 subjects (10 in each arm) will be randomly selected to take part in the GTI substudy. The GT image capturing, which is based on the principle of optical triangulation, is non-contact and non-invasive to the patient, and it aims to clarify differences in the wound healing pattern and association to clinical and patient-centred outcomes between the two groups. These subjects will undergo all study visits in the same way as other subjects but will attend the additional imaging analysis at some study visits, as well as at some additional visits as outlined below. The image capture, analysis and measurements will be performed in the selected participants at baseline, day 1 and day 5 after non-surgical treatment and 3 months and 9 months follow-ups, and, in the surgical group, additional measurements will be performed at 1 week and 1 month after surgery.

#### Patient-reported outcomes (PROMS)

A substantial body of evidence suggests that the presence of periodontitis has a considerable effect on the quality of life of affected individuals [33]. This effect will be assessed by measuring patient-reported outcome measures (PROMs). Patient-reported outcomes will be collected using validated patient questionnaires (Oral Impact on Daily Performance (OIDP), EuroQol five-dimension (EQ-5D) scale and global ratings for periodontal health and quality of life [QoL]) [34–36] at baseline and at the 3, 9 and 15 months follow-ups. In addition, subjects allocated to the control group (MIST) will provide PROMS at the 1-week review appointment after the surgical procedure. Furthermore, the subjects allocated to the GTI subgroup will provide PROMS at the 1 day and 5 days post-treatment visits.

#### Statistical analysis plan

The objectives and outcomes are to investigate whether MINST is not inferior to M-MIST in terms of intrabony defect depth healing in patients with periodontitis after 15 months follow-up. These outcomes are measured as:

- Primary outcome: radiographic intrabony defect depth change
- Secondary outcomes:
  - o PPD and CAL change (in millimetres)
  - o Inflammatory markers and growth factors in GCF
  - o Bacterial detection associated with presence of intrabony defects
  - o Gingival inflammation and healing (as measured by GTI in a subset of 10 test and 10 control patients)
  - o PROMs.

The sample size calculation was based on the assumption that the proposed modified MINST protocol is an acceptable alternative to the M-MIST protocol (non-inferiority), with the advantage of reduced costs and morbidity. A non-inferiority margin of 1 mm is considered to be the largest difference that is acceptable between MINST and M-MIST (a lower threshold than the expected difference in bone gain between regenerative surgeries and open flap debridement in intrabony defects) [12] for MINST to be adopted in clinical practices because of the associated advantages of MINST. Single-flap approaches such as M-MIST have previously been reported to lead to radiographic bone gains from 1.8 ± 1.2 mm [37] to 2 mm [38] to 3.5 ± 1.0 mm [14] in small trials. MINST has been reported to lead to 2.4 ± 2.1 mm radiographic bone gain in a retrospective study [17]. Therefore, we assume that the two protocols lead to the same bone gain for the sample size calculation. Accounting for a 10% drop-out rate and using a pooled standard deviation of 1.27, recruiting *n* = 66 participants will be sufficient to confer 90% power to reject the inferiority null hypothesis. Should the true mean bone gain difference between MINST and M-MIST be 0.19 mm in favour of M-MINST, the study would retain a statistical power of 80%. The primary outcome analysis will compare the intrabony defect depth between treatment groups at 15 months using a general linear analysis of covariance (ANCOVA) model with treatment as a factor and the corresponding baseline value as a covariate and adjusting for pre-specified prognostic baseline factors (age, FMPS, BMI, defect angle, tooth mobility). For the primary efficacy endpoint, non-inferiority of M-MIST to MINST could be claimed if the lower limit of the 95% confidence interval (for the difference in mean change of radiographic intrabony defect depth change) is greater than − 1.0 mm. The primary outcome analysis will be the per-protocol analysis; the intention-to-treat analysis will be considered as the sensitivity analysis. For other non-inferiority endpoints the per-protocol analysis will also be considered the main analysis. The superiority endpoint will be analysed on an intention-to-treat basis. Subject-based and site-based analyses will be conducted. The PROM analysis will be based on linear or logistic regression (for continuous/categorical variables) comparing PROM scores or categories of scores at follow-up (3, 9 and 15 months post-intervention) between the two groups, accounting for the respective scores at baseline. We will also look at the minimally important difference for PROMs where applicable, such as in the case of the OIDP, and whether any difference between the two groups is clinically meaningful.

#### Data management

All data will be entered in a dedicated secure database application with a secure web connection (REDCap). A customised REDCap project will be set up and will be used to cover all data capture for the study. Different levels of access will be set up for the different end users/study team delegates. Data will be proofed for entry errors before being locked and exported for analysis.

#### Randomisation and allocation concealment

Following the baseline visit, all participants enrolled in the study will be randomly assigned to one of the two treatment groups and whether or not to be included into the GTI substudy; that is, individual-level randomisation to MINST, M-MIST, MINST + GTI or M-MIST + GTI will be performed using a 2:2:1:1 allocation ratio. Random permuted block randomisation with block sizes 6 and 12 will be employed. The centralised online randomisation service ‘Sealed Envelope’ with web front-end will be used ensuring allocation concealment. No minimisation or stratification is planned.

#### Blinding

Due to the nature of the intervention, only blinding of all outcome examiners is possible. Both participants and clinicians administering treatment will be unblinded. Members of the Trial Steering Committee and other study team members will remain blinded to treatment allocation until the randomisation code is broken (after the last follow-up data are recorded and the database locked).

#### Potential risks or burdens for research participants and how to minimise them

No risks or burdens are expected from the basic periodontal examination and treatment. Minor pain or discomfort may follow the subgingival debridement and can be easily controlled by using a 0.2% chlorhexidine digluconate solution rinse and, if needed, paracetamol 2 × 500 mg up to four times a day for the first 2 days. The surgical interventions are also standard routine procedures and will be carefully planned and performed under local anaesthesia. The following post-operative regime will be followed in the first and second weeks following the completion of the treatment in order to minimise the patient’s discomfort and risk of complications: post-operative pain will be controlled with paracetamol if required, and all patients will be instructed to discontinue locally tooth brushing at the surgical site to minimise trauma and to rinse with 0.2% chlorhexidine digluconate two times/day for the first 2 weeks.

Adverse events (AEs) which may be related to periodontal surgery, GTI images and other dental or non-dental (including sample collection) procedures may be recorded on the AE log based on the study medical team assessments. AEs which are assessed by the study medical team as deviating, i.e. in severity, intensity and frequency, from potentially expected AEs will be recorded on the AE log. Potentially expected AEs are commonly reported AEs following non-surgical and surgical periodontal therapy such as gingival bleeding, bruising and swelling in the first 2–3 days post-therapy and increase in tooth sensitivity in the first 1–2 weeks post-therapy.

Any serious adverse event (SAE) occurring to a research participant will be reported to the sponsor within 24 h of learning of the event and to the Research Ethics Committee (REC) within 15 days if the Chief Investigator (CI) considers the event to be related to the study research procedures and unexpected.

Risk considerations for this study include the following:

1. *Safety of patients’ sensitive data*: We will mitigate this by following the current information governance regulations. All patients’ data held will be coded and anonymised, encrypted and physically stored in a protected (locked or in a cloud) data location.
2. *Safety of dental and other procedures*: All dental or other procedures (including radiographs and sample collection) proposed in this study fall within the scope of mainstream periodontal treatment. Furthermore, all clinical treatment will be performed by an appropriately trained clinician. In case of any unfavourable AE or adverse reaction, the study medical team will assess its severity, relatedness and potential consequences to the study patients and will make an informed decision as per the affected patient’s suitability to remain in or be exited from the study. Should the patient be exited from the study, he/she will be offered appropriate periodontal or dental treatment and any other support as needed.
3. *Risk of not being able to complete the study due to lack of patient recruitment*: Careful analysis of patient flow and current experience from other studies has estimated that 66 suitable patients could be screened and enrolled in 6–7 months. If not, the study recruitment period will be extended, or other contingencies will be sought after including opening more sites if needed.

Reporting of any suspected expected or unexpected SAEs will be communicated to the study sponsor following standard research governance protocols as well as to the REC approving this study. The study monitoring will be performed by the clinical research facility manager and/or the study coordinator in pre-determined intervals as per the monitoring and management plan. Annual and safety reports to the REC will be submitted annually, and the REC will be notified of any corrective actions or mitigations and contingencies planned or implemented as necessary.

#### Data handling and record keeping

Barts Health National Health Service (NHS) Trust will collect information from patients in order to contact them when needed and to make sure that relevant information about the study is recorded for their care. Barts Health NHS Trust will keep identifiable information about patients in this study for 20 years after the study has finished. Information related to participants will be kept confidential and managed in accordance with the General Data Protection Regulation (GDPR) (EU) 2016/679 Data Protection Act, NHS Caldicott Principles, the Research Governance Framework for Health and Social Care and the conditions of REC Approval.

#### Monitoring and auditing

Internal regular monitoring visits will take place to ensure that all trial-related activities are conducted according to the trial protocol and that the data were recorded, analysed and accurately reported according to the protocol, the sponsor’s standard operating procedures (SOPs), Good Clinical Practice (GCP) and the applicable regulatory requirement(s). Internal audits may be conducted by a representative of the sponsor, the QMUL or the funder (Barts Charity) for this study if deemed necessary. The QMUL COCR will internally monitor and manage the study on behalf of the sponsor (QMUL). The trial management and monitoring plan (including the frequency of monitoring visits), the level of source data verification (SDV) and percentage of consent monitoring or the level of proportional review for data transfer from source documents to CRFs will be set up at the beginning of the trial by COCR-delegated personnel. Any arrangement for monitoring and auditing the conduct of the study will be critically examined to ensure it complies with the relevant parties’ allocation of responsibilities as set out in the Research Governance Framework.

#### Trial committees

Focus groups including study investigators, nurses, patients and members of the public were organised in the planning stages of the study and then to review study documents including the patient information sheet and consent form. A study-specific Trial Management Group (TMG) will meet ideally every month including the study CI as well as the relevant co-investigators and other team members. TMGs are aimed at discussing the routine management of this research project and any clinical or other type of deviations from the study protocol, sponsor’s SOPs, GCP or the applicable regulatory requirement(s). A Trial Steering Committee (TSC) including the trial statistician, the study CI, the imaging scientist, co-investigators or collaborators, the study data management and coordinating team, a member of the public and a patient representative will convene ideally every 6 months. Study focus groups including one or two study investigators, study coordinator or nurse and a panel of four or five individuals selected from hospital patients and the public will be held annually starting from the set-up phase, in order to advise on study design and delivery.

#### Dissemination

Results of this study are likely to be disseminated through scientific dental journals with open access policies and international periodontal conferences. Public access to the full protocol, participant-level dataset and statistical code will be provided upon reasonable request. We also plan to disseminate our research findings in a language that needs to be easily understood by a lay person attending the local public engagement meetings, through leaflet distribution and our webpages and with the use of patient forums. Dissemination of project outcomes will also take place across the larger Barts Health communities and stakeholders, setting up future research pathways, support and collaborative agreements.

## Discussion

This study will produce data on the efficacy and potential applicability of a modified MINST protocol for the treatment of periodontal intrabony defects. If shown to be non-inferior (in terms of radiographic and clinical defect reductions) to the tested surgical approach, MINST, being a less invasive option, might be suggested potentially as an alternative to the use of surgical procedures. Results of the study will be published in peer-reviewed journals and published at international conferences following completion of all study analyses.

### Trial status

Protocol version number 1.2 dated 13 September 2018 has received ethics approval in November 2018 as stated above. Patient recruitment started at the beginning of 2019 and is expected to be completed by late 2019 or early 2020, after which we will only follow up on existing study patients. Any necessary amendments to the study protocol will be submitted for ethics approval and, upon approval, the relevant changes will be made to the ClinicalTrials.gov database and the *Trials* publication.

## Data Availability

The final database will be accessible for all members of the research team (the authors of this protocol). The datasets generated will not be publicly available. However, following study completion, study data will be made available in de-identified form by the corresponding author for any reasonable request.

## Abbreviations

AE: Adverse event
BOP: Bleeding on probing
CAL: Clinical attachment loss/level
CI: Chief Investigator
CRF: Case Report Form
CS: Control site
DS/RS: Dentine/root sensitivity
FMBS: Full-mouth bleeding score
FMPS: Full-mouth plaque score
GCF: Gingival crevicular fluid
GCP: Good Clinical Practice
GT: Geometric thermal
GTI: Geometric thermal imaging
IS: Intrabony site
MIST: Minimally invasive surgical therapy
M-MIST: Modified minimally invasive surgical therapy
NHS REC: National Health Service Research Ethics Committee
NSPT: Non-surgical periodontal therapy
OHR-QoL: Oral health-related quality of life
OIDP: Oral Impact on Daily Performance
PPD: Probing pocket depth
PROM: Patient-reported outcome
RCT: Randomised controlled trial
REC: Research Ethics Committee
SAE: Serious adverse event
SDV: Source data verification
SOP: Standard operating procedure
TMG: Trial Management Group
TSC: Trial Steering Committee
